# 

**Published:** 1825-04-01

**Authors:** 


### Contents

OF

No. 4. Vol. II. (NEW SERIES) for APRIL, 1825.

[*No.* 20, *ANALYTICAL SERIES.*]

ART. I.

A Practical Treatise on Hæmorrhoids, Strictures, and other Important Diseases of the Rectum and Anus, &c. By GEO. CALVERT, Esq. .. .. .. .. .. .. .. .. .. 273

II.

OPHTHALMIA.

1. Mr. THOMAS HEWSON on Ophthalmia accompanying the Secondary Forms of Lues Venerea .. .. .. . .. } 305

2. Dr. O'HALLORAN on Acute and Chronic Ophthalmia .. }

III.

Recherches Anatomico-Pathologiques sur la Médécine Pratique, &c. By M. TACHERON, M.D... .. .. .. .. . 326

IV.

Dr. DUNCAN, Jun. on Diffuse Inflammation of the Cellular Texture, &c. .. .. .. .. .. .. .. .. .. .. 335

V.

Mr. Richard Phillips' Translation of the New Pharmacopœia, with Notes, &c... .. .. .. .. .. .. .. .. .. 356

VI.

A Treatise on Mental Derangement. By FRANCIS WILLIS, M.D. 360

VII.

A Treatise on Moxa, as applicable more especially to Stiff Joints, &c. By JAMES BOYLE, Esq. .. .. .. .. .. .. 377

### VIII.

### PRACTICE OF MEDICINE.

1. Dr. Uwins' Compendium of Theoretical and Practical Medicine .. .. .. .. .. .. .. .. .. .. } 382
2. Dr. Dawson's Nosological Practice of Physic, embracing Physiology .. .. .. .. .. .. .. .. .. .. }

### IX.

Dr. Blundell's Physiological and Pathological Researches .. 400

### X.

Dr. Buchan on Symptomatology. .. .. .. .. .. .. 413

### XI.

Dr. Cox on Acute Rheumatism .. .. .. .. .. .. .. .. 428

### XII.

### Quarterly Periscope

OF

### PRACTICAL MEDICINE;

BEING

*THE SPIRIT OF THE MEDICAL JOURNALS,*

FOREIGN AND DOMESTIC.

### I. PHYSIOLOGY.

1. M. Martinet on the Vital Power of Resistance (Idiosyncrasy) 433
2. Dr. Roesler on Resuscitation in suspended Animation.. .. 440
3. On the Etiology of Goître, Cretinism, &c. .. .. .. .. 442

### II. PATHOLOGY.

1. M. Andral on Fatal Dyspnœa. .. .. .. .. .. .. 444

2. Dr. Chapman on Inflammation of Veins. .. .. .. .. 447

3. M. Nauche on Irritation and Inflammation .. .. .. .. 451

4. Mr. North on a Peculiar Spasm in Children.. .. .. .. 452

5. Post-mortem Appearances in the Case of Dr. Henning .. 457

6. Fatal Asthma from Affection of the Glottis .. .. .. .. 458

7. Remarkable Case of Intus-Susception .. .. .. .. .. 460

8. M. Cayol's Hospital Reports on Fever, &c. &c. .. .. .. 460

9. Tympanitic State of the Pericardium .. .. .. .. .. 465

### III. SURGERY.

1. Supposed Fracture of the Basis Cranii . .. .. .. .. .. 466

2. Mr. Yeatman on the Treatment of Hernia .. .. .. .. .. 467

3. Dr. Robertson on Iritis. .. .. .. .. .. .. .. .. .. .. 468

4. Mental Imbecility from Injury of a Nerve .. .. .. .. .. 469

5. Mr. Melin on Ophthalmia .. .. .. .. .. .. .. .. .. 470

6. Mr. Snell's Case of Anchylosis of the Jaws . .. .. .. 472

7. The Recto-Vesical Operation of Lithotomy . .. .. .. .. 473

Dr. Muter's Description of the Operation .. .. .. .. .. 474

8. Dr. Martland on Staphyloma treated by Tapping . .. .. 475

9. Dr. Barnes' Account of the Knife-Eater's last Illness.. .. 476

10. Dr. Thompson on Retention of Urine.. .. .. .. .. .. 477

11. Mr. Money on Paracentesis Capitis in Hydrocephalus . .. 478

12. History of Paracentesis Thoracis, with Cases (Retrospective) 479

### IV. THERAPŒIA.

1. Emetics in Hæmatemesis .. .. .. .. .. .. .. .. .. 481

2. Dr. Hosack's Hospital Reports—

Amenorrhœa, Intermittent Fevers, Phthisis, Chronic Rheumatism, Syphilis .. .. .. .. .. .. .. .. .. .. 482

3. Dr. Davies on Puerperal Peritonitis .. .. .. .. .. 483

4. Dr. M‘ Dowel on Buchu Leaves.. .. .. .. .. .. 485

5. Mr. Creighton on Tartar Emetic Ointment in Epilepsy .. 486

6. Mr. Ferrall's Case of Poisoning by Nitre .. .. .. .. 487

7. Dr. Barker on Sulphate of Quinine .. .. .. .. .. 488

8. Dr. Borthwick on the Effects of Constipation .. .. .. 489

9. Dr. Beatty's Case of Severe Affection of the Head .. .. 490

10. Dr. Begbie on Datura Stramonium .. .. .. .. .. 491

11. Peculiar Convulsion in Children.. .. .. .. .. .. 493

12. Dr. O'Beirne on Tobacco in Dysentery .. .. .. .. 495

13. Professor Recamier and Dr. Fievée on Hydrophobia.. .. 497

14. Dr. Godelle on Compression in Ascites .. .. .. .. 500

15. Dr. Fisher on Hydrocephalus cured by Mercury.. .. .. 501

16. Boiling Bath in Chronic Rheumatism.. .. .. .. .. 502

### V. MIDWIFERY.

1. On Puerperal Irritability .. .. .. .. .. .. .. .. 503

2. Dr. Ollivier on Ascites complicated with Pregnancy .. .. 506

3. Preternatural Presentation, with Remarks .. .. .. .. .. 507

4. Dreadful Puerperal Fever at Vienna .. .. .. .. .. 509

5. On Irritable Uterus .. .. .. .. .. .. .. .. .. .. 512

6. Dr. Musgrave's Case of Repeated Abortions .. .. .. 513

7. M. Baudeloque's Substitute for the Cæsarean Section.. .. 522

8. Dropsy complicated with Pregnancy .. .. .. .. .. 523

9. M. Dugés on Puerperal Neuritis . .. .. .. .. .. .. 523

10. Case of Separation of the Pubes .. .. .. .. .. .. 526

VI. MEDICAL JURISPRUDENCE.

Culpable Abortion, Trial for .. .. .. .. .. .. .. .. 528

### XIII.

ANALECTA MINORA, OF MINOR PERISCOPE.

1. Extinction of Fire .. .. .. .. .. .. .. .. .. .. 530

2. Italian Remedies .. .. .. .. .. .. .. .. .. .. .. 530

3. Pressure on the Perineum on Delivery .. .. .. .. .. 530

4. M. Bally's Report on Certain Medicines .. .. .. .. 530

    1. Sulphate of Quinine .. .. .. .. .. .. .. .. .. 530

    2. Acetate of Morphine .. .. .. .. .. .. .. .. .. 531

    3. Extract. Lactucarii, .. .. .. .. .. .. .. .. .. 531

    4. Nitrate of Potash in Dropsy .. .. .. .. .. .. .. 531

5. Mr. Bushell on Flowers of Colchicum .. .. .. .. .. 531

6. Baynton's Method of Treating old Ulcers, not new .. .. 532

7. Mr. Battley on the Volatile Oil of Cubebs .. .. .. .. 532

8. Intra-Uterine Vociferation .. .. .. .. .. .. .. .. 532

### XIV.

Bibliographical Record .. .. .. .. .. .. .. .. .. .. 533

### XV.

EXTRA-LIMITES.

I. Mr. Wise on the less frequent Use of the Trephine .. .. 536

II. Dr. Gregory's Report from the Small Pox and Vaccination Hospital .. .. .. .. .. .. .. .. .. .. .. .. .. 551

III. Mr. Waterworth on the Sand-Rock Spring, Isle of Wight .. 555

IV. Crown Life Assurance Company .. .. .. .. .. .. .. 556

V. State of the Westminster Medical Society, Great Marlbro' Street .. .. .. .. .. .. .. .. .. .. .. .. .. 558

VI. Mr. Reeve's Tooth-Instrument .. .. .. .. .. .. .. 560

VII. Dr. Smith on the Duty of a Coroner respecting Medical Evidence .. .. .. .. .. .. .. .. .. .. .. 561

### XVI.

INTELLIGENCE, CORRESPONDENCE, &c.

Establishment of a General Infirmary at Chichester .. .. .. 564

Factitious Mineral Waters at Brighton .. .. .. .. .. .. 564

Death of Dr. Chisholm .. .. .. .. .. .. .. .. .. .. 565

Additional Subscribers .. .. .. .. .. .. .. .. .. .. 566

